# MicroRNA-338-3p helps regulate ovarian function by affecting granulosa cell function and early follicular development

**DOI:** 10.1186/s13048-023-01258-3

**Published:** 2023-08-26

**Authors:** Ziwen Xu, Tongwei Zhang, Jingyi Hu, Junya Zhang, Guang Yang, Jiahuan He, Huihui Wang, Ran Jiang, Guidong Yao

**Affiliations:** 1 Center for Reproductive Medicine, The First Affiliated Hospital of Zhengzhou University, Zhengzhou, China; 2 Henan Key Laboratory of Reproduction and Genetics, The First Affiliated Hospital of Zhengzhou University, Zhengzhou, China

**Keywords:** microRNA, Ovarian function, Granulosa cell, Oocyte quality, Follicular development

## Abstract

**Background:**

Follicular development in mammalian ovaries is a complex and dynamic process, and the interactions and regulatory-feedback loop between the follicular microenvironment, granulosa cells (GCs), and oocytes can affect follicular development and normal ovary functions. Abnormalities in any part of the process may cause abnormal follicular development, resulting in infertility. Hence, exploring the pathogenesis of abnormal follicular development is extremely important for diagnosing and treating infertile women.

**Methods:**

RNA sequencing was performed with ovarian cortical tissues established in vitro. In situ-hybridization assays were performed to study microRNA-338-3p (miR-338-3p) expressed in GCs and oocytes. In vitro culture models were established with GCs and neonatal mouse ovaries to study the biological effects of miR-338-3p. We also performed *in viv*o experiments by injecting adeno-associated virus vectors that drive miR-338-3p overexpression into the mouse ovarian bursae.

**Results:**

Sequencing analysis showed that miR-338-3p was expressed at significantly higher levels in ovarian cortical tissues derived from patients with ovarian insufficiency than in cortical tissues derived from patients with normal ovarian function; miR-338-3p was also significantly highly expressed in the GCs of patients with diminished ovarian reserve (P < 0.05). In situ-hybridization assays revealed that miR-338-3p was expressed in the cytoplasm of GCs and oocytes. Using in vitro culture models of granulosa cells, we found that miR-338-3p overexpression significantly suppressed the proliferation and oestradiol-production capacity of GCs (P < 0.05). In vitro culture models of neonatal mouse ovaries indicated that miR-338-3p overexpression suppressed the early follicular development in mouse ovaries. Further analysis revealed that miR-338-3p might be involved in transforming growth factor β-dependent regulation of granulosa cell proliferation and, thus, early follicular development. Injecting miR-338-3p-overexpression vectors into the mouse ovarian bursae showed that miR-338-3p down-regulated the oocyte mitochondrial membrane potential in mice and disrupted mouse oestrous cycles.

**Conclusion:**

miR-338-3p can affect early follicular development and normal ovary functions by interfering with the proliferation and oestradiol production of GCs. We systematically elucidated the regulatory effect of miR-338-3p on follicular development and the underlying mechanism, which can inspire new studies on the diagnosis and treatment of diseases associated with follicular development abnormalities.

**Supplementary Information:**

The online version contains supplementary material available at 10.1186/s13048-023-01258-3.

## Background

The ovarian follicle represents the basic unit of the mammalian ovary that performs reproductive and secretory functions. A woman is born with a finite number of primordial follicles in her ovarian follicle bank and after puberty, a population of primordial follicles begins to develop periodically under the influence of endogenous hormones. Eventually, however, less than 1% of the primordial follicles can develop into mature follicles and ovulate, and most of the primordial follicles undergo follicular atresia [1].

Women experience a sharp decline in their ovarian reserve of primordial follicles and a gradual decline of ovarian function after the age of 40. A premature decrease in the number of recruitable follicles in the ovaries or delayed follicular development can lead to a diminished ovarian reserve (DOR). Devine et al. conducted an epidemiological survey on DOR and found that the prevalence of DOR increased from 19 to 26% from 2004 to 2011 among US patients treated with assisted reproductive technology (ART) procedures [2]. The ovarian reserve is crucial for the maintenance of fertility in women, and the capacity of follicular development determines the state of the ovarian reserve [3]. Premature ovarian insufficiency (POI) is a continuum of declining ovarian function that occurs in woman under the age of 40 that is manifested by menstrual disturbance (amenorrhea, oligomenorrhea, or polymenorrhoea) with elevated levels of gonadotropins and decreased levels of oestrogen. Patients with POI experience reduced fertility or a near loss of fertility and low oestrogen levels, which increase the patient’s long-term risk of developing osteoporosis and cardiovascular diseases; thus, it is especially important to limit the pathogenesis of POI. The aetiology of POI is highly heterogeneous and the currently known causes mainly include genetic factors, immune factors, and iatrogenic factors (among others), with genetic factors being considered an important etiological factor for POI. The coding regions of genes associated with POI have been extensively studied, and non-coding RNAs have gained increasingly wider attention among researchers in recent years. Some data have suggested that miRNAs are widely expressed in mammalian ovaries and that their expression levels change with the progression of follicular development [4]. These findings indicate that miRNAs play major roles in follicular development.

An ovarian follicle mainly consists of an oocyte located centrally within the follicle, GCs surrounding the oocyte, and theca cells and follicular fluid in the follicular lumen. The proliferation and secretory activities of GCs play crucial roles in follicular development and directly affect the status of follicular development [5]. Previous data revealed that granulosa cell apoptosis and a decreased number of granulosa cell layers can cause abnormal changes in the ratio of sex hormones in the follicular fluid, resulting in an undersupply of nutrients to the oocyte and disrupted intercellular signal transduction, which eventually leads to the inhibition of follicular development and, consequently, a poor ovarian reserve [6]. The aetiology of DOR has yet to be fully elucidated, but genetic factors, environmental factors, ovarian damage caused by ovarian surgery/chemotherapy/radiotherapy, or abnormal expression of transforming growth factor (TGF, a regulator of follicular development) can lead to abnormal ovarian follicle development, excessive depletion of primordial follicle reserves, inhibited development of primary and secondary follicles, accelerated follicular atresia, and the consequent occurrence of DOR [7]. Similarly, abnormalities during any stage of follicular development can result in POI, such as an excessively small ovarian reserve of primordial follicles, accelerated follicular atresia, and abnormalities in the recruitment or functions of follicles. In-depth studies on the functions of GCs and follicular development can help reveal the molecular mechanisms underlying the pathogenesis of diseases associated with abnormalities in follicular development, such as DOR and POI.

MicroRNAs (miRNAs) comprise a class of small (22–25 nucleotide) non-coding RNAs that serve regulatory roles [8]. miRNAs are initially transcribed as long single-stranded primary miRNAs (pri-miRNAs), and these pri-miRNAs are cleaved in the cell nucleus by the Drosha (ribonuclease)–DGCR8 complex to generate small stem–loop RNA structures (65–70 base pairs in length) called precursor miRNAs (pre-miRNAs). Subsequently, the stem–loop pre-miRNAs are exported to the cytoplasm through the nuclear pores by the nuclear export protein, Exportin 5, and then cleaved by the enzyme, Dicer, to release small-molecule RNA dimers in the cytoplasm. After further processing, the RNA dimers become mature miRNAs that bind to the intracytoplasmic AGO protein to form the miRNA-induced silencing complex and perform their biological functions [9]. Although the transcripts of the miRNA genes cannot be translated to proteins, they are widely known to regulate biological activities at the post-transcriptional level [10]. miRNAs primarily inhibit gene expression in two ways: (1) miRNAs bind to the 3′-untranslated region (3′-UTR) of target messenger RNAs (mRNAs) and induce silencing of the target genes via full pairing with complementary bases. (2) miRNAs bind to the 3′-UTRs of mRNAs via partial pairing of complementary bases to repress gene expression [11]. In addition, a study by Vasudevan et al. suggested that some miRNAs such as miR-369-3p, let-7, and cxcr can positively regulate gene expression by promoting the translation of mRNAs into proteins during the G1 phase of the cell cycle [12].

MiRNAs can be applied for the diagnosis, monitoring and prognosis of cancer and endocrine diseases considering that they are stable in human tissues and body fluids, are highly conserved, and are not easily degraded by ribonucleases [13]. In the field of reproductive science, miRNAs are also being used as emerging molecular markers to assess ovarian functions, endometrial receptivity, and embryo quality [14]. As mentioned above, the occurrence of DOR is closely associated with the proliferation and the apoptotic state of granulosa cells. The results of previous studies suggested that miRNAs can affect the hormonal production, proliferation, differentiation, and apoptosis of GCs by regulating the expression levels of steroidogenic acute regulatory (StAR) protein, cytochrome P450 19A1 (CYP19A1) gene, transforming growth factor β1 (TGFβ1) - drosophila mothers against decapentaplegic (Smads) signalling pathway, as well as the activity of the phosphatidyl inositol 3-kinase (PI3K)/protein kinase B (Akt)/mammalian target of rapamycin (mTOR) signalling pathway [15, 16].

In this study, we found that miR-338-3p was expressed at significantly high levels in ovarian cortical tissues from patients with POI via RNA sequencing. In addition, we established in vitro culture of GCs and mouse ovaries and performed in vivo mouse experiments to investigate the impact of miR-338-3p on the proliferation and hormonal production of granulosa cells, early follicular development, oocyte quality, and the oestrous cycle.

## Materials and methods

### Sample collection and cell culture

Sixty-eight infertile patients who underwent in vitro fertilization (IVF)- or intracytoplasmic sperm injection (ICSI)-based ART procedures at the Center for Reproductive Medicine in the First Affiliated Hospital of Zhengzhou University were recruited and enrolled in this study. B-mode ultrasound-guided transvaginal oocyte retrieval was performed by introducing the puncture needle through the posterior fornix of the vagina to obtain the patients’ follicular fluid. Primary human GCs were then isolated and purified by density-gradient centrifugation, and approximately 50% of GCs have the ability to survive. Except for the need to explore research related to different types of granulosa cells derived from different ovarian function, other experiments are conducted to pool granulosa cells from multiple patient sources to reduce the possible impact on the results due to individual differences in patients. The normal control group was composed of patients who had normal ovarian function, but experienced infertility caused by tubal factors or male factors. Patients who previously received surgical treatment for ovarian or pelvic disorders; underwent pre-implantation genetic diagnosis for familial hereditary diseases; or had systemic diseases, endometriosis, or thyroid dysfunction were excluded from this study. Patients who met the following criteria were included in the DOR group: ≤45 years of age, Anti-Mullerian Hormone: 0.5–1.1 µg/L, Antral Follicle Count: 5–7, basic Follicle Stimulating Hormone: 10–40 IU/L, normal chromosomal karyotype.

Primary human GCs and KGN cells (a human granulosa-like tumour cell line) were cultured in DMEM/F12 (Gibco, Waltham, USA) medium supplemented with 10% charcoal/dextran-treated foetal bovine serum (FBS; HyClone, Logan, USA) and 1% penicillin and streptomycin sulphate (HyClone) at 37℃ with 5% CO_2_ and saturated humidity. The primary GCs cultured in vitro were irregular polygonal adherent growth cells. During the experiments, primary GCs were treated with recombinant TGF-β1 dissolved in 4 mM HCl containing 0.1% bovine serum albumin (BSA), whereas the control group was treated with an equal volume of 4 mM HCl containing 0.1% BSA.

### Real-time polymerase chain reaction (PCR) analysis

Total RNA was extracted from primary human GCs using TRIzol (Invitrogen, Carlsbad, USA), and RNase-free water (Solarbio, Beijing, China) was added to dissolve the precipitated RNA before repeated blowing for an even mixture. The concentration and purity of the RNA obtained were measured using a NanoDrop instrument (Thermo Fisher Scientific, Waltham, USA). The concentration of RNA were 595.1-623.4 ng/µL, and the OD260/280 and OD260/230 of RNA were 1.95–1.97 and 2.10–2.24, respectively. The Hairpin-it™ miRNA qRT-PCR Quantitation Kit (GenePharma, Shanghai, China) was used to reverse transcribe the extracted total RNA into complementary DNA (cDNA) by incubation at 25℃ for 30 min, at 42℃ for 30 min, and at 85℃ for 5 min. Real-time fluorescence-based quantitative PCR were then performed to amplify and quantify the reverse-transcribed cDNA using the by incubation Hairpin-it™ miRNA qRT-PCR Quantitation Kit (GenePharma) in a 7500 Real-Time PCR System (Bio-Rad Laboratories, Hercules, USA) at 95℃ for 3 min, followed by 40 cycles of 95℃ for 12 s and 62℃ for 40 s. The relative expression levels of miR-338-3p were determined by the 2^−ΔΔCT^ method: ΔCT = ΔCT target – ΔCT reference; -ΔΔCT = – (ΔCT sample – ΔCT control). U6 RNA was detected as the internal reference control gene. The primers used for the PCR were listed in Supplementary Table S1.

**Fluorescence**in situ**hybridization (FISH)**.

After the human primary GCs were attached to slides and fixed with 4% paraformaldehyde at room temperature for 20 min, a gene pen was used to draw circles on the cell slides. Then, 20 µg/ml proteinase K (Servicebio, Wuhan, China) was added dropwise in the circles and the cells were digested for 5 min, after which prehybridization solution (Servicebio) was added dropwise, and the samples were incubated for 1 h at 37℃. Next, hybridization solution containing miR-338-3p probes (8 ng/µl; probe sequence: 5′-CAACAAAATCACTGATGCTGGA-3′) was added dropwise and hybridization was allowed to proceed overnight at 37℃. On the next day, the mixture was blocked in 0.1% BSA diluted with phosphate-buffered saline (PBS; Servicebio) at room temperature for 30 min after the hybridization solution was removed with saline sodium citrate (SSC; Servicebio). A Cy3-labelled anti-DIG antibody (Jackson Laboratories, West Grove, USA) was then added dropwise before the mixture was incubated at 37℃ for 50 min. Subsequently, 4,6-diamidino-2-phenylindole (DAPI; Cell Signaling Technology, Danvers, USA) was added under dark conditions, the samples were incubated for 8 min, and images were collected under an inverted fluorescence microscope Axio Observer (Carl Zeiss, Oberkochen, Germany).

### Granulosa cell transfections

GCs were expanded in 6-well plates and transfected when the cell density reached 70-80%. To perform the transfections, 125 µl of Opti-MEM (Thermo Fisher Scientific) was first mixed with 7.5 µl of Lipofectamine RNAiMAX (Invitrogen) in a Eppendorf (EP) centrifuge tube, the mixture was vortexed, and the tube was centrifuged. In separate tubes, 125 µl of Opti-MEM was mixed with a set volume of stock solution containing miR-338-3p mimics, an miR-338-3p inhibitor, or the corresponding negative control (GenePharma, Shanghai, China), after which the EP tubes were vortexed and centrifuged. Subsequently, equal volumes of both types of solutions (solutions containing the transfection reagent and one of the indicated nucleotides) were evenly mixed, centrifuged, and allowed to stand for 10 min. After the medium in the 6-well plates was replaced with fresh DMEM/F12 medium, the transfection mixture was added dropwise and evenly to the 6-well plates. At 6 h post-transfection, the medium in each 6-well plate was discarded and replaced with DMEM/F12 medium containing 10% FBS. miR-338-3p overexpression or inhibition in GCs was verified by real-time PCR (Supplementary Figure S1).

**Fig. 1:**
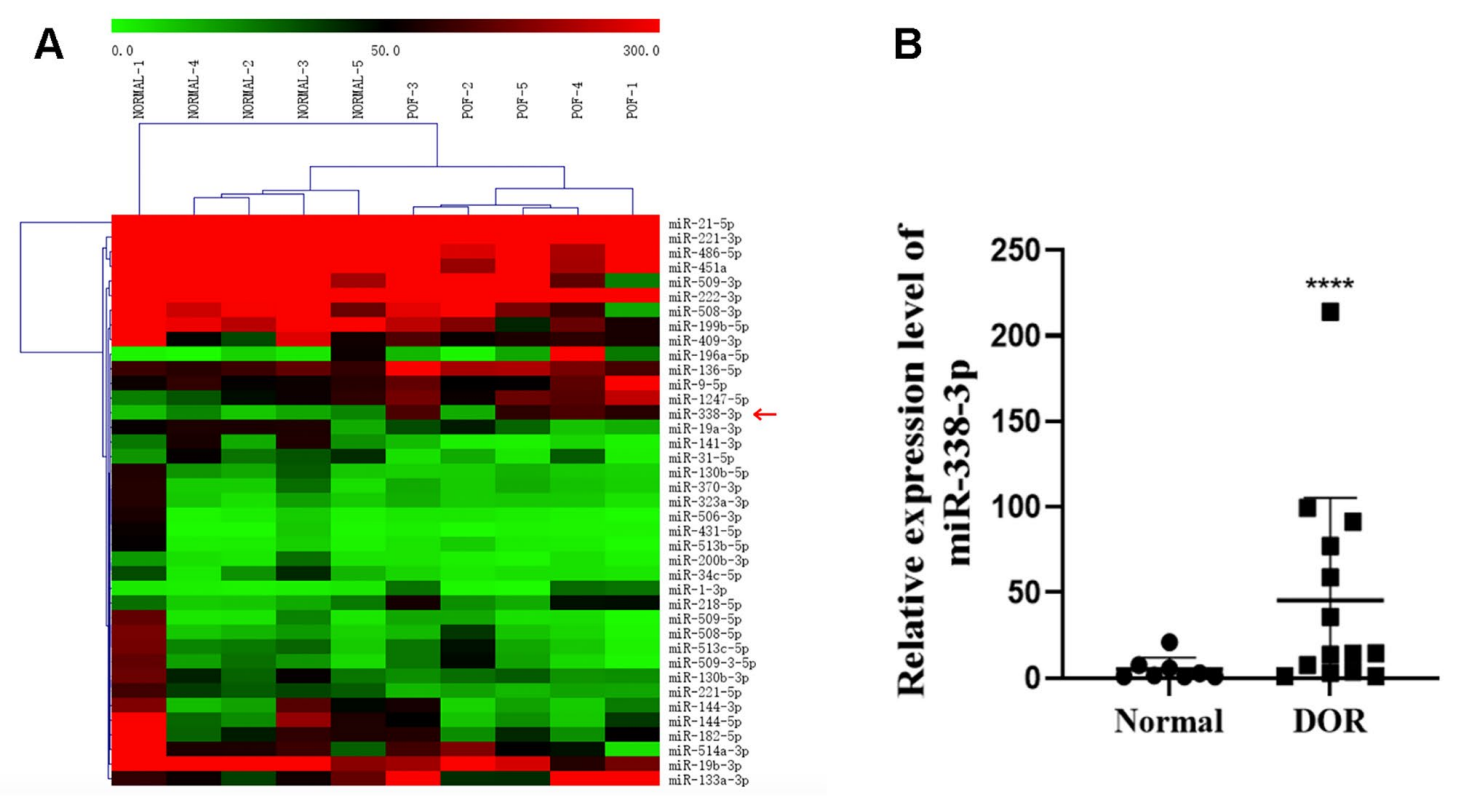
miR-338-3p expression in ovarian cortical tissues and granulosa cells. (**A**) Next-generation sequencing was performed to analyse miRNA-expression profile in the ovarian cortical tissues derived from patients with POI or normal ovarian function, and a clustering-analysis map was plotted for the differentially expressed miRNAs. The red and green blocks indicate differentially expressed genes in ovarian cortical tissues derived from POI patients that were up-regulated or down-regulated, respectively. The X-axis corresponds to the ovarian cortical tissue samples derived from patients with normal ovarian function or POI, and the Y-axis corresponds to the differentially expressed miRNAs. (**B**) Follicular fluid samples were collected from patients with normal ovarian function or a low ovarian reserve. The primary GCs were purified by density-gradient centrifugation and total RNA was extracted using TRIzol. After the extracted total RNA was reverse transcribed, real-time PCR was performed to detect the relative expression levels of miR-338-3p. U6 RNA was detected as the internal reference. ****P < 0.0001

### Cell-proliferation analysis

Cells were evenly inoculated into 96-well cell culture plates at a density of approximately 4000 cells/well. The cells were transfected when they entered logarithmic growth phase on the next day. A solution for detecting cell proliferation was prepared by mixing 10 µl of Cell-Counting Kit-8 (CCK-8; Boster Biological Technology, Wuhan, China) with every 100 µl of DMEM/F12 medium. Then, 110 µl of the resulting solution was added to each well after thorough mixing, and each 96-well plate was placed in a CO_2_ incubator for 2 h of further incubation. Next, the optical density at 450 nm (OD_450_) was measured with a Varioskan Flash microplate reader (Thermo Fisher Scientific).

### Oestradiol measurements

To determine the capacity of primary human GCs to secrete oestradiol (E_2_), the cells were evenly inoculated into 6-well plates at a density of 1 × 10^6^ cells/well. When the cell density reached approximately 70%, the cells were transfected with miR-338-3p mimics or an miR-338-3p inhibitor. At 6 h post-transfection, the original growth medium was replaced with culture medium containing 10^− 7^ M testosterone (Sigma, St Louis, USA), and the cells were cultured for another 48 h. Subsequently, the culture medium was collected and the supernatant was harvested by centrifugation. The E_2_ concentration in the supernatant was measured in a Roche Diagnostics Cobas 6000 instrument (Roche Diagnostics) using an Electrochemiluminescence Immunoassay Kit (Roche Diagnostics, Rotkreuz, Switzerland).

### Western blotting

Primary human GCs were lysed with high-efficiency cell lysis buffer (Solarbio) and centrifuged at 10,000 × *g* and 4℃, in order to harvest the protein samples from the supernatants. The OD values of the standard protein and test protein samples were measured at 595 nm using a microplate reader, with Quick Start™ Bovine Serum Albumin (Bio-Rad Laboratories) serving as the standard for the protein assays. The proteins were separated by sodium dodecyl sulphate polyacrylamide gel electrophoresis and transferred to polyvinylidene difluoride (PVDF) membranes. Subsequently, the PVDF membranes were blocked in Tris-buffered saline and tween 20 (TBST, CWBIO, Beijing, China) containing 5% skimmed milk powder for 1 h, primary antibodies were added, and the membranes were incubated overnight at 4℃. On the next day, the PVDF membranes were washed with TBST and incubated with appropriate secondary antibodies in 5% skimmed milk on a shaker at room temperature for 1 h. After washing the membranes with TBST, the immunoreactive bands were detected using enhanced chemiluminescent substrates (Bio-Rad Laboratories) and the images of chemiluminescent blots were captured with a ChemiDoc MP imager (Bio-Rad Laboratories). The primary antibodies used in this study included antibodies against B-cell lymphoma 2 (Bcl-2; 1:10000 dilution; Abcam, Cambridge, UK), protein 53 (p53; 1:1000; Cell Signaling Technology), aromatase (1:500; Abcam), and glyceraldehyde-3-phosphate dehydrogenase (GAPDH; 1:5000; Abcam). The secondary antibodies used in this study included a polyclonal goat anti-mouse IgG antibody (1:5000; Abcam) and a polyclonal goat anti-rabbit IgG antibody (1:5000; Abcam).

### Immunofluorescence

Primary human GCs were inoculated on cell slides and transfected with miR-338-3p mimics, an miR-338-3p inhibitor, or the corresponding control treatment when the cell density reached approximately 70%. After 48 h of further cell culture, the culture medium in each 6-well plate was discarded. The cells attached to the slides were fixed with 4% paraformaldehyde at room temperature for 20 min, after which PBS containing 0.5% Triton X-100 (PBST; Solarbio) was used to permeabilize the cells for 20 min. Subsequently, the cell slides were blocked with PBS containing 5% BSA for 40 min and incubated overnight at 4℃ with anti-Ki-67 antibodies diluted in PBS supplemented with 5% BSA (1:1000, Cell Signaling Technology). After washing the slides three times in PBST, they were incubated with a secondary Alexa Fluor 488 AffiniPure Goat Anti-Rabbit IgG antibody (Jackson ImmunoResearch, West Grove, USA) for 30 min under dark conditions. Finally, the cell nuclei were stained with 1 µg/ml DAPI diluted in PBS (Sigma) for 10–15 min. After completing the counterstaining, the images were observed and collected under a fluorescence microscope (Carl Zeiss).

Each mouse was administrated an intraperitoneal injection of 0.1 ml of Pregnant Mare Serum Gonadotropin (10 international units (IUs)/ml; Solarbio), followed 48 h later by an intraperitoneal injection of 0.1 ml 10 IU/ml hCG (Solarbio). Sixteen hours after the second injection, the mice were sacrificed by cervical dislocation to harvest the fallopian tubes and collect the cumulus–oocyte complexes (COCs) under a microscope. The collected COCs were then treated with hyaluronidase (Aibei Biotechnology, Nanjing, China) to remove the cumulus cells and further cultured in growth medium for 1–2 h. Finally, the oocytes were stained using the Mitochondrial Membrane Potential Assay Kit (JC-1; Beyotime, Shanghai, China) according to the manufacturer’s instructions, and the images were observed and collected under a fluorescence microscope (Carl Zeiss).

### In vitro culture of new-born mouse ovaries

Neonatal D1 and D4 female CD1 (ICR) mice (Charles River Laboratories, Beijing, China) were sacrificed by cervical dislocation, and an abdominal wall and skin incision was made in the ventral midline to fully expose the abdominal viscera. After the Y-shaped uterus and kidneys above the uterus were located, the adipose tissue at the junction between the kidneys and the upper segment of the uterus was separated and placed in PBS (Solarbio) at 37℃, and the neonatal mouse ovaries were isolated under a stereomicroscope (Nikon, Tokyo, Japan). The isolated ovaries were divided into three groups: the fresh-control group, the adenovirus-infected control group, and the group transfected with an miR-338-3p-overexpression adenoviral vector. The neonatal mouse ovaries in the fresh-control group were placed in 4% paraformaldehyde for fixation, followed by subsequent haematoxylin and eosin (HE) staining immediately after isolation. The mouse ovaries in the adenovirus-infected control group and the group transfected with the miR-338-3p-overexpression adenoviral vector were cultured at 37℃ with 5% CO_2_ and saturated humidity in α-MEM (Gibco, Waltham, USA) supplemented with 10% FBS and 1% penicillin and streptomycin sulphate. Adenoviral vectors (adv-EGFP or adv-miR-338-3p) at a titre of 5 × 10^10^ plaque-forming units/ml were added to the in vitro culture system for neonatal mouse ovaries. At 6 h post-transfection, the original growth medium was replaced with normal culture medium, the ovaries were culture for an additional 48 h, and then the cells were re-transfected with adv-EGFP or adv-miR-338-3p for 6 h. The growth medium was then replaced with normal culture medium, and the cells were cultured for an additional 48 h. After the completion of ovarian culture, the ovaries were fixed with 4% paraformaldehyde and HE staining was performed.

### HE staining and ovarian follicle recognition

To confirm the effect of miR-338-3p on follicular development, we first fixed the treated neonatal mouse ovaries with 4% paraformaldehyde overnight at 4℃. After fixation, the ovarian tissues were immersed and dehydrated in ethanol, and then embedded in paraffin wax. The paraffin block was continuously sectioned at a thickness of 5 μm, followed by dewaxing, rehydration and HE staining. Follicles at different maturation stages were identified based on a previously reported method [17]. The criteria for ovarian follicle recognition were as follows: primordial follicles were identified by an oocyte surrounded by a single layer of flattened granulosa cells; primary follicles were identified by an oocyte surrounded by a single layer of cuboidal granulosa cells; secondary follicles were identified by an oocyte surrounded by two or more layers of cuboidal granulosa cells, without an observable follicular antrum; and antral follicles were identified by an oocyte surrounded by two or more layers of granulosa cells, with a clearly observable follicular antrum.

### In situ injection of mouse ovaries

Eight-week-old CD1 mice (Charles River) were weighed and anaesthetized by intraperitoneal injection with normal saline solution containing 2% pentobarbital sodium (Sigma; 45 mg/kg body weight). The anaesthetized mice were then placed in a prone position on a sterile drape. After the surgical sites were located on both sides of the lumbar spine, shaved, and disinfected with 75% ethanol to expose the surgical field of vision. Next, a 1 cm skin incision was made in the surgical field, and ophthalmic forceps were used to locate the ovaries in the surgical field and gently lift up the adipose tissue surrounding the ovaries, which exposed the ovarian bursa. Subsequently, 10 µL of AAV-EGFP or AAV-miR-338-3p was injected into the ovarian bursa. After the injection, the injection site was allowed to stand for 3 min so that the injected fluid fully infiltrated into the ovary. Subsequently, the ovary was gently pushed back into the abdominal cavity and gentamicin was dripped into the surgical incision before the incision was sutured layer by layer. After surgery, the mice were allowed to wake up and recover from anaesthesia in a warm and dry environment, and they were returned to the rearing cage after they resumed activity.

### Oestrous cycle identification

The oestrous cycle of the mice was monitored by examining exfoliated cells from the vagina. A thin sterile cotton swab was moistened with normal saline solution, gently inserted 0.5 cm into the vaginal cavity of each mouse, and slowly rotated 360°. The vaginal contents adhering to the thin cotton swab were spread evenly on a glass slide to prepare a vaginal smear, which was subsequently air-dried. The air-dried smear was then immersed in 95% ethanol for 5 min, stained with haematoxylin solution (Solarbio) for 8 min, rinsed with water for 30 s, immersed in ethanol containing 19% HCl for 10 s, rinsed with water for 1 min, dehydrated with 95% ethanol for 3 min, stained with eosin solution (Solarbio) for 30 s, rinsed with water for 20 s, and finally dehydrated in 95% ethanol for 2 min. After the staining procedures were completed, the vaginal smears were observed under a microscope for histological changes to identify the different phases of the oestrous cycle [18].

### Statistical analysis

SPSS software (version 20.0) was used for statistical data analysis. Normally distributed continuous variables were expressed as the mean ± standard deviation. The parametric Student’s t-test was performed to compare two groups, and one-way analysis of variance (ANOVA) or two-way repeated-measures ANOVA was performed to compare three or more groups. The chi-squared test was performed to compare categorical variables. A P value of < 0.05 indicates differences of statistical significance. Each experiment was independently repeated at least three times.

## Results

### miR-338-3p was highly expressed in ovarian cortical tissues of patients with POI and in GCs from patients with DOR

To investigate the regulatory effects of miRNAs on the occurrence and development of POI, we analysed the miRNA-expression profiles in ovarian cortical tissues from five patients with POI and five patients with normal ovarian function. The results revealed 39 significantly differentially expressed miRNAs between the cortical tissues derived from patients with POI and patients with normal ovarian function (*P* < 0.05) (Fig. 1A), including miR-196a-5p, miR-133a-3p, miR-1-3p, miR-338-3p, miR-1247-5p, miR-218-5p, miR-9-5p, miR-136-5p, miR-19b-3p, miR-222-3p, miR-21-5p, miR-514a-3p, miR-221-3p, etc. (Supplementary Table S2). miR-338-3p expression was significantly higher in ovarian cortical tissues derived from patients with POI than in cortical tissues derived from patients with normal ovarian function (*P* < 0.0001, fold-change > 2).

To further study the correlation between miR-338-3p expression and ovarian function, we collected follicular fluid from 8 patients with normal ovarian function and 14 patients with DOR, and isolated primary granulosa cells from the collected follicular fluids. Reverse transcriptase-quantitative polymerase chain reaction (RT-qPCR) analysis was performed to measure the relative expression levels of miR-338-3p in granulosa cells. The expression levels of miR-338-3p in GCs derived from patients with DOR were significantly higher than in those derived from control patients with normal ovarian function (*P* < 0.05; Fig. 1B).

### The expression and localization of miR-338-3p in primary human ovarian GCs and mouse ovaries

**Fig. 2:**
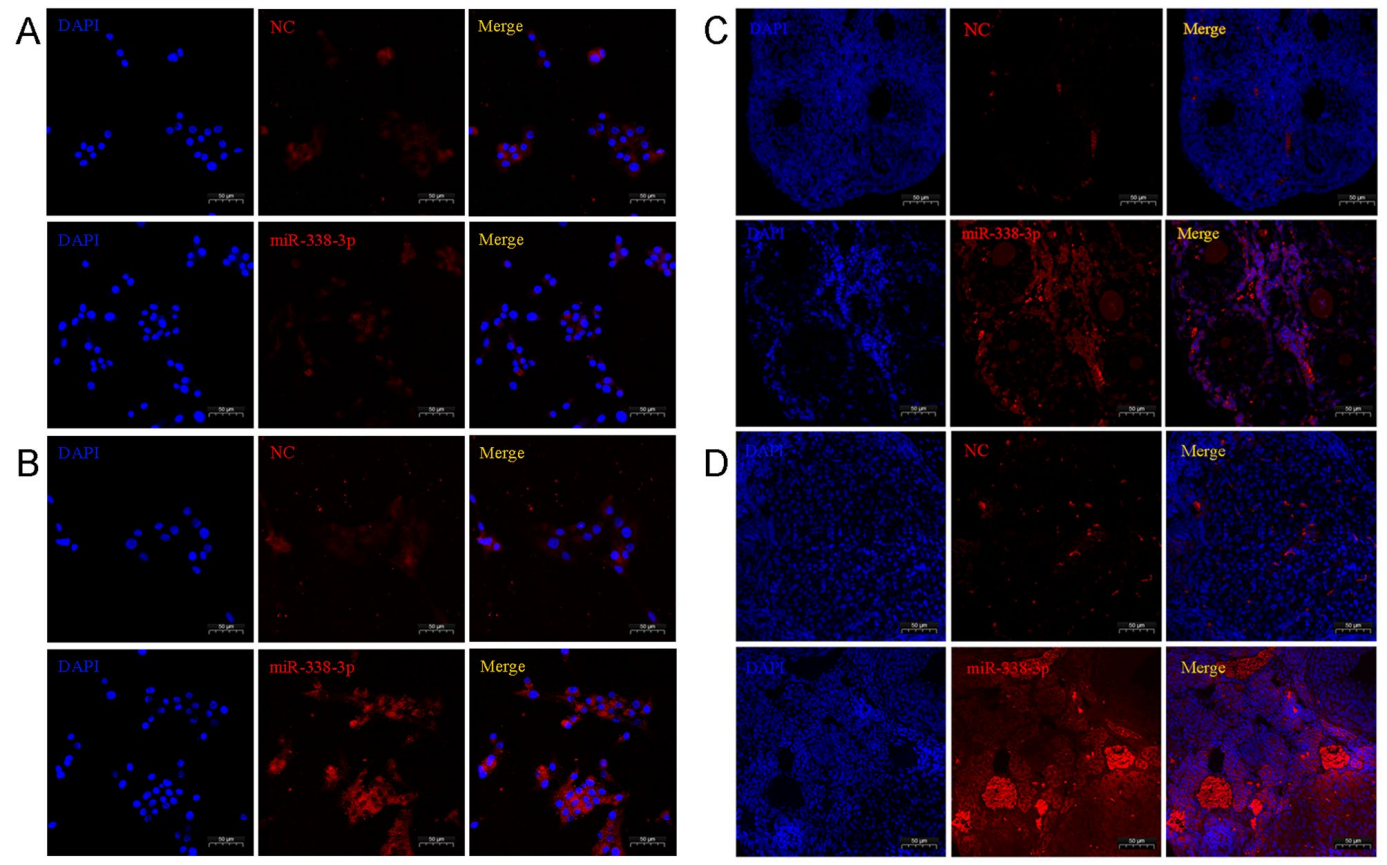
Expression and localization of miR-338-3p in human primary GCs and mouse ovaries. (A&B) Primary human GCs in human follicular fluid from patients with normal ovarian function (**A**) or DOR (**B**) were purified by density-gradient centrifugation, inoculated on cell slides in 6-well plates, and cultured for 24 h. After routine fixation of the cells, in situ hybridization was performed to detect the expression and localization of miR-338-3p. (**C**&**D**) After routine fixation and embedding of the obtained normal mouse ovaries (**C**) or POI mouse ovaries (**D**), in situ hybridization was performed to detect the expression and localization of miR-338-3p in the tissues. The blue signals reflect nuclear staining by DAPI. The red signals represent the NC or miR-338-3p signals, as indicated. Scale bars: 50 μm

To further investigate the impact of miR-338-3p on follicular development and ovarian function, we performed FISH to analyse miR-338-3p expression and localization in primary human GCs and mouse ovaries. We observed that miR-338-3p was expressed in primary human GCs and was mainly located in the cytoplasm; miR-338-3p expression was not observed in the cell nuclei. Moreover, the expression level of miR-338-3p in granulosa cells from patients with DOR was significantly higher than that in the control group (Fig. 2A, B). Because hsa-miR-338-3p and mmu-miR-338-3p have the same sequence (5′-UCCAGCAUCAGUGAUUUUGUUG-3′; https://www.mirbase.org/), we further analysed miR-338-3p expression and localization in mouse ovarian tissues. We found that miR-338-3p was mainly expressed in the GCs and oocytes of mouse ovarian follicles and that miR-338-3p was also located in the cytoplasm of mouse GCs and oocytes; miR-338-3p expression was not observed in the cell nuclei. Besides, the expression level of miR-338-3p in the ovaries of POI model mice was significantly higher than that of the control group (Fig. 2C, D).

### Overexpression of mir-338-3p suppressed the proliferation of GCs and their capacity to secrete oestradiol

**Fig. 3:**
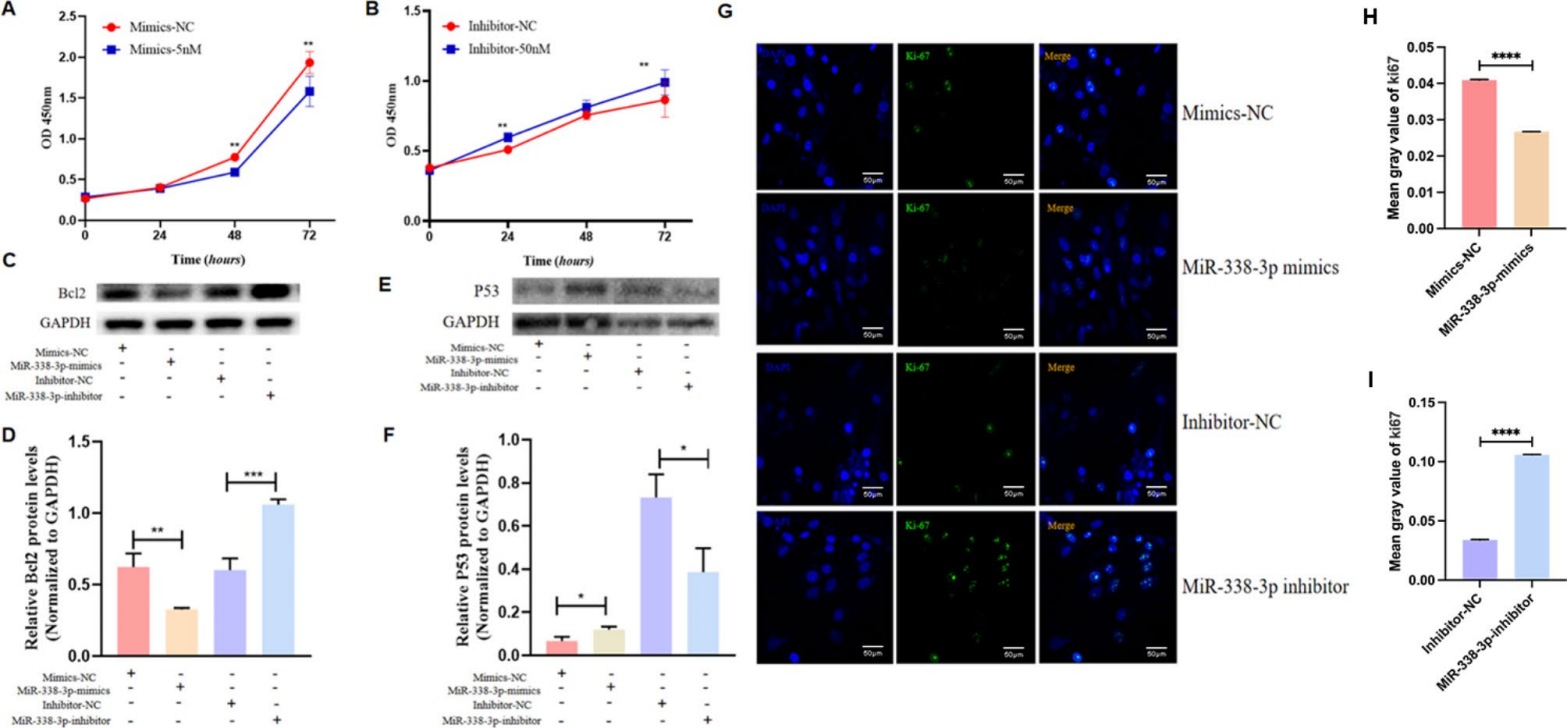
miR-338-3p overexpression suppressed granulosa cell proliferation. (**A**) The KGN cell line was seeded in 96-well plates and transfected with miR-338-3p mimics or negative-control (NC) mimics, the OD_450_ values were measured at 0, 24, 48, and 72 h using the CCK-8 Kit, and cell-growth curves were plotted. (**B**) The KGN cells were transfected with miR-338-3p inhibitors or NC inhibitors; the OD_450_ values were measured at 0, 24, 48, and 72 h; and the cell-growth curves were plotted. (**C**-**F**) Human primary ovarian GCs were transfected with miR-338-3p mimics/inhibitor or their NCs, in order to manipulate the expression of miR-338-3p. After 48 h of further culture, the cellular proteins were extracted and the western blotting analysis was performed to detect the protein-expression levels of Bcl2 (**C**) and p53 (**E**) in each group, and to identify inter-group differences in terms of Bcl-2 protein expression (D) and p53 protein expression (**F**). The GAPDH protein expression was used as the internal reference control. (**G**) Primary human ovarian GCs were inoculated on cell slides, and the cells were transfected for 48 h with miR-338-3p mimics/inhibitor or their NCs to manipulate the expression of miR-338-3p. Subsequently, the cells were fixed for immunofluorescence staining to analyse the expression of the proliferation-associated nuclear antigen, Ki-67. The blue fluorescence indicates nuclear staining by DAPI. The green fluorescence represents the proliferation-associated nuclear antigen, Ki-67. Scale bar: 50 μm. The histogram shows the ratio of target antibody/DAPI mean fluorescence intensity (**H**, **I**). *P < 0.05, **P < 0.01, ***P < 0.001, ****P < 0.0001

As mentioned above, miR-338-3p was detected in GCs and oocytes and was highly expressed in the ovarian cortical tissues derived from patients with POI and GCs derived from patients with DOR. These results suggest that miR-338-3p might help regulate granulosa cell functions. KGN GCs were transfected with miR-338-3p mimics to induce miR-338-3p overexpression or miR-338-3p inhibitors to down-regulate miR-338-3p expression; cell-proliferation curves were plotted using the CCK-8 method. The results suggest that miR-338-3p overexpression significantly inhibited KGN cell proliferation (*P* < 0.01; Fig. 3A) and that miR-338-3p down-regulation significantly promoted KGN cell proliferation (*P* < 0.01; Fig. 3B). Western blotting analysis showed that miR-338-3p overexpression was accompanied by lower Bcl-2 expression, whereas inhibiting miR-338-3p expression was accompanied by significantly elevated Bcl-2 expression (*P* < 0.01; Fig. 3C, D). miR-338-3p overexpression was accompanied by significantly elevated p53 protein levels, whereas inhibiting miR-338-3p expression was accompanied by significantly lower p53 protein levels (*P* < 0.05; Fig. 3E, F). Granulosa cell proliferation was further analysed by detecting the intracellular expression of Ki-67. The results revealed that the intranuclear Ki-67-positivity rates were significantly lower in the miR-338-3p-overexpression group than in the control group, whereas miR-338-3p down-regulation was accompanied by significantly increased Ki-67 positivity rates (*P* < 0.0001; Fig. 3G-I).

**Fig. 4:**
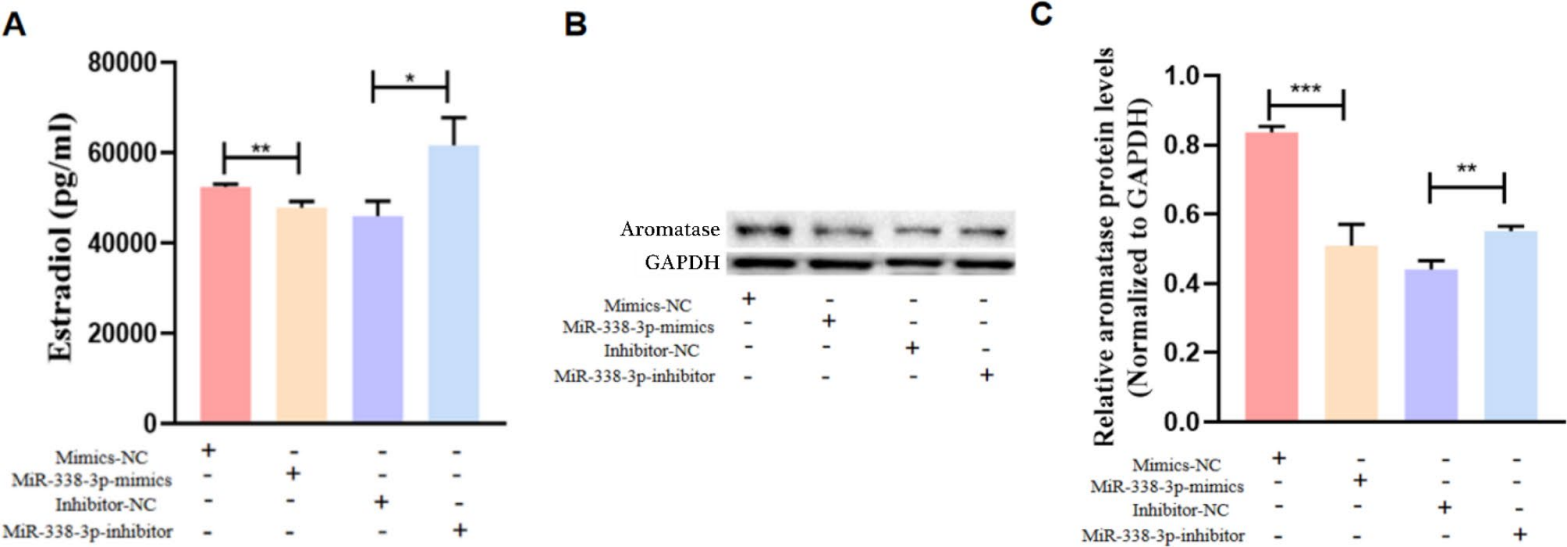
miR-338-3p overexpression suppressed the E_2_-production capacity of primary human granulosa cells. (**A**) The GCs were inoculated in 6-well plates and expanded by routine culture for 24 h. Next, the original culture medium was replaced with cell-starvation medium, followed by 24 h of further culture. The cells were then transfected with miR-338-3p mimics/inhibitor to manipulate the expression level of miR-338-3p and cultured for 48 h, after which the culture supernatant was collected to determine the E_2_ levels by performing an electrochemiluminescence immunoassay. (**B**) Primary human GCs were transfected with miR-338-3p mimics/inhibitor to manipulate miR-338-3p expression, the cells were cultured for 48 h, and western blotting analysis was performed to determine the expression level of aromatase. (**C**) Image J software was used to perform grayscale analysis of the aromatase protein bands. The bar chart displays the ratio of the grayscale value of the aromatase protein to that of the GAPDH protein. *P < 0.05, **P < 0.01, ***P < 0.01

To determine the impact of miR-338-3p on the oestrogen-production capacity of granulosa cells, we detected oestradiol concentrations in the culture medium of primary GCs after altering the expression of miR-338-3p. Overexpressing miR-338-3p was accompanied by significantly lower E_2_ levels in the culture supernatant (*P* < 0.01), whereas inhibiting miR-338-3p expression was accompanied by significantly elevated E_2_ levels in the culture supernatant (*P* < 0.05; Fig. 4A). Overexpressing miR-338-3p in GCs significantly down-regulated the expression of aromatase (*P* < 0.01), a rate-limiting enzyme involved in oestradiol biosynthesis), whereas down-regulating miR-338-3p expression significantly elevated aromatase expression (*P* < 0.01; Fig. 4B, C).

### Overexpressing miR-338-3p suppressed early follicular development in neonatal mouse ovaries culture in vitro

Our preliminary experiments suggested that miR-338-3p expression was up-regulated in GCs from patients with ovarian hypofunction, which inhibited granulosa cell proliferation and oestradiol secretion from granulosa cells. Therefore, we hypothesized that miR-338-3p might interfere with follicular development by regulating granulosa cell function and thereby induce ovarian hypofunction. To test this hypothesis, we altered miR-338-3p expression in in vitro-cultured neonatal mouse ovaries and analysed the developmental statuses of follicles at different maturation stages.

**Fig. 5:**
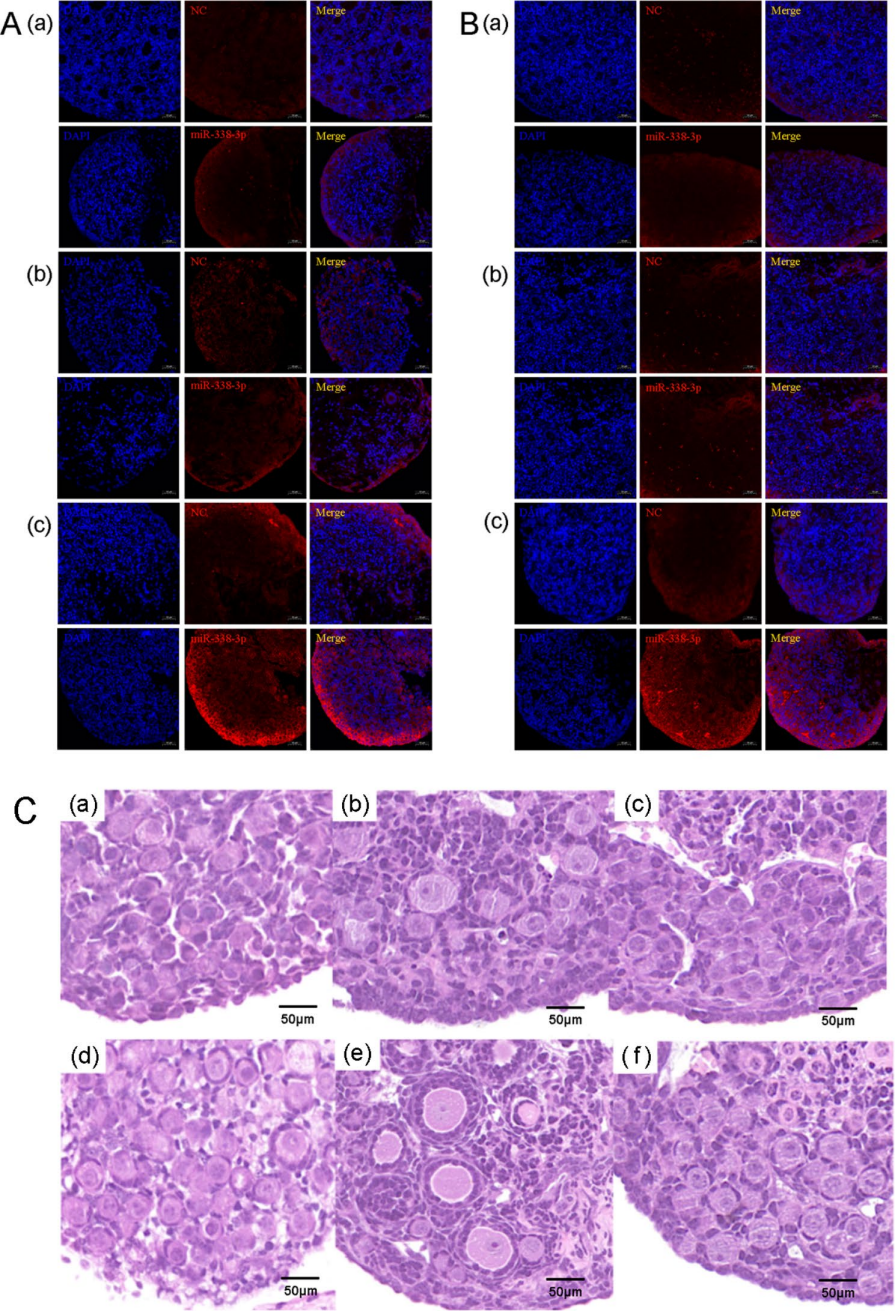
The effect of miR-338-3p overexpression on follicular development in neonatal mouse ovaries cultured in vitro. (**A**&**B**) After routine fixation and embedding of the obtained D1 mouse ovaries (**A**) or D4 mouse ovaries (**B**), in situ hybridization was performed to detect the expression and localization of miR-338-3p in the tissues. (**a**): fresh-control group. (**b**): adenovirus-control group. (**c**): miR-338-3p-overexpression group.The blue signals reflect nuclear staining by DAPI. The red signals represent the NC or miR-338-3p signals, as indicated. (**C**): (**a**, **d**) The fresh-control group consisted of neonatal (D1, D4) mouse ovaries that were obtained, immediately fixed, routinely embedded, and then HE-stained. (**b**, **e**) The adenovirus-control group consisted of neonatal (D1, D4) mouse ovaries that were cultured in vitro, treated with adv-EGFP for 96 h, and then fixed. (**c**, **f**) The miR-338-3p-overexpression group (i.e. the experimental group) consisted of neonatal (D1, D4) mouse ovaries that were cultured in vitro, treated with adv-miR-338-3p for 96 h to induce miR-338-3p overexpression, and then fixed. Scale bars: 50 μm

Firstly, we validated the overexpression effect of miR-338-3p in the neonatal mouse ovaries by FISH (Fig. 5A, B). After ovaries from neonatal D1 mice were cultured in vitro for 96 h, the follicles were morphologically normal and were capable of continued development (Fig. 5C(a), (b)), indicating that the in vitro culture system for neonatal D1 mouse ovaries can support the survival and continued development of mouse ovarian follicles. Similarly, ovaries from neonatal D4 mice also survived and continued to develop in the culture system (Fig. 5C(d), (e)). Compared with the fresh-control group, similar follicular morphologies and structures were maintained in the D1 neonatal mouse ovaries in the miR-338-3p-overexpression group, but the development of most primordial follicles was arrested (Fig. 5C(a), (c)). In contrast, the follicles in the negative-control group maintained their capacity for continued development, and the single layers of flattened GCs surrounding the oocytes mostly developed into multiple layers of cuboidal GCs (Fig. 5C(b)). These results suggest that miR-338-3p overexpression suppressed the primordial-to-primary follicle transition. HE staining of neonatal (D4) mouse ovaries revealed that the oocytes in the miR-338-3p-overexpression and fresh-control groups were still mostly surrounded by a single layer of GCs (Fig. 5C(d), (f)). In contrast, the follicles in the neonatal (D4) mouse ovaries of the negative-control group notably exhibited a capacity for continued development: the number of granulosa cell layers around the oocytes increased and secondary follicles developed (Fig. 5(e)). These results indicate that miR-338-3p impeded both the primordial-to-primary follicle transition and the primary-to-secondary follicle transition.

### TGF-β promoted granulosa cell proliferation by down-regulating miR-338-3p expression

**Fig. 6:**
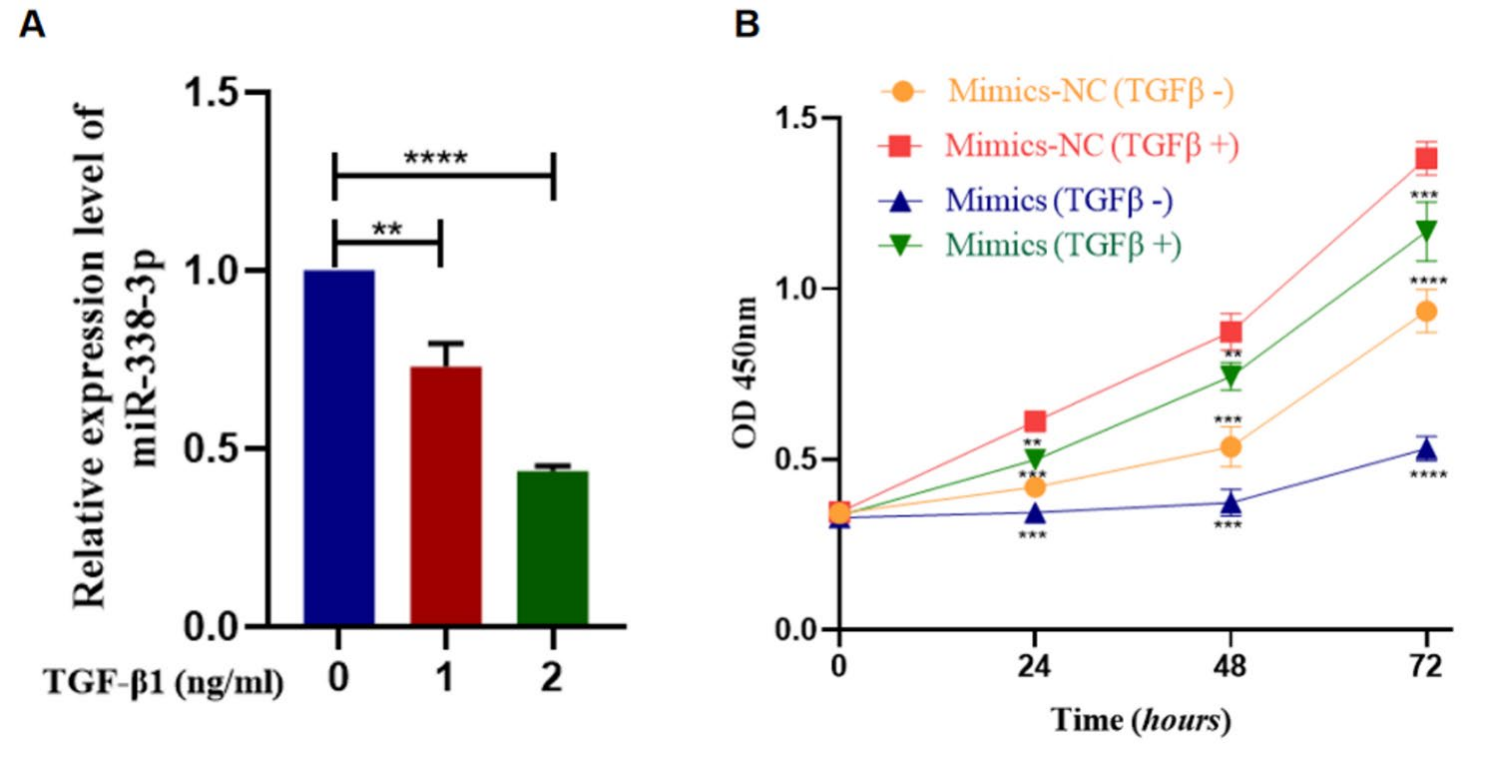
miR-338-3p was involved in TGF-β1-dependent regulation of granulosa cell proliferation. (**A**) After the primary human GCs were routinely cultured in vitro for 24 h, the original culture medium was replaced with cell-starvation medium and the cells were cultured an additional 24 h. The cell-starvation medium was then replaced with culture medium supplemented with different concentrations of TGF-β1 (0, 1, or 2 ng/ml), and the cells were treated in this manner for 6 h. After the treatment was completed, total RNA was extracted from the GCs and real-time PCR was performed to analyse miR-338-3p expression. U6 RNA was detected as the internal reference. (**B**) The GCs were routinely seeded in 96-well plates and then transfected with miR-338-3p mimics/NC when the cell density reached approximately 70%. At 6 h post-transfection, the original culture medium was replaced with culture medium supplemented with TGFβ1 or control growth medium. The OD_450_ values were measured after 0, 24, 48, and 72 h by performing cell-viability assays, and cell-growth curves were plotted. The “mimics” group represents the miR-338-3p-overexpression group transfected with miR-338-3p mimics, and the “mimics-NC” group represents the NC group transfected with NC mimics. **P < 0.01; ***P < 0.001; ****P < 0.0001

Previous reports suggested that TGFβ plays a major role in regulating early follicular development by regulating the proliferation and differentiation of granulosa cells, hormonal production, and theca cell function. Hence, we hypothesized that miR-338-3p might also help mediate the TGF-β signalling pathway and regulate follicular development, thereby affecting the ovarian reserve. In view of this hypothesis, we tested whether TGF-β can affect miR-338-3p expression and whether inhibiting miR-338-3p can overcome the regulatory effect of TGF-β on granulosa cell proliferation. TGF-β1 significantly suppressed miR-338-3p expression in GCs (*P* < 0.01), and the inhibitory effects were more prominent at higher TGF-β1 concentrations (Fig. 6A). Cell-viability assays indicated that TGF-β1 significantly promoted granulosa cell proliferation and that miR-338-3p significantly suppressed granulosa cell proliferation. In addition, the GCs in the miR-338-3p-overexpression group treated with TGF-β1 exhibited significantly weaker proliferation than the group treated with TGF-β1 alone (Fig. 6B). These results indicate that miR-338-3p suppressed the pro-proliferative effect of TGF-β1 on granulosa cells.

### Overexpressing miR-338-3p in vivo affects the number of mouse follicles, the quality of mouse oocytes and the oestrous cycle

**Fig. 7:**
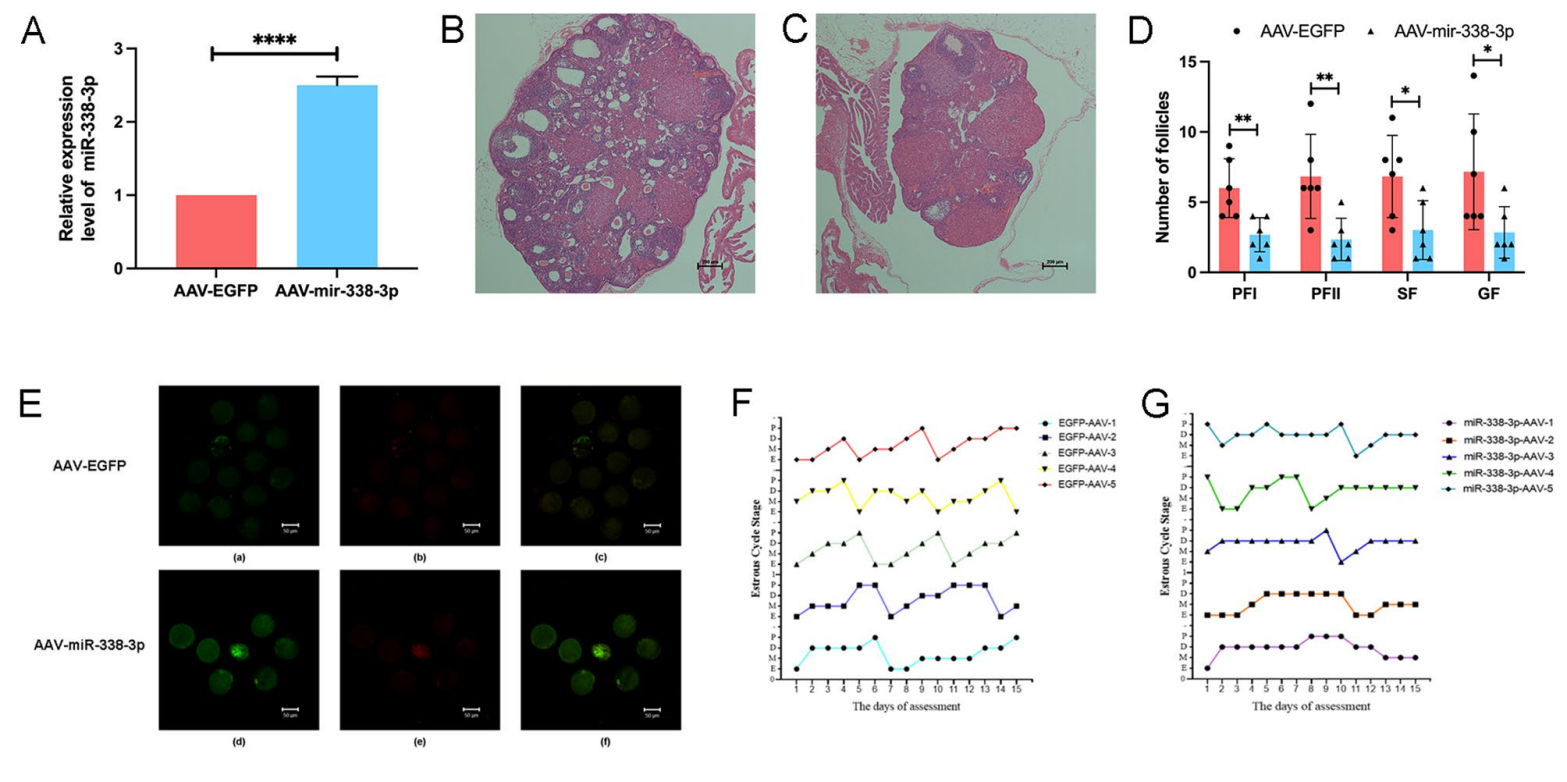
Effects of miR-338-3p on the number of follicles, the oocyte mitochondrial-membrane potential and the oestrous cycle. (**A**) The control group consisted of 2-month-old female mice receiving injections of EGFP-adeno-associated virus (AAV) into their ovarian bursae and the miR-338-3p-overexpression group consisted of mice receiving injections of miR-338-3p-AAV. After 35 days of injection, total RNA from mouse ovaries was extracted respectively from the control group and the miR-338-3p-overexpression group and real-time PCR was performed to analyse miR-338-3p expression. U6 RNA was detected as the internal reference. (**B**-**D**) At 35 days post-injection, mouse ovaries were obtained and HE staining was used to analyze the differences in the number of follicles at all levels between the control group (**B**) and the miR-338-3p overexpression group (**C**). (**E**) mouse oocytes were harvested and stained with the JC-1 dye to analyse the oocyte mitochondrial-membrane potential in the control group (**a**-**c**) and the miR-338-3p-overexpression group (**d**-**f**). The green mitochondria showed maximum fluorescence emission between 515 and 530 nm, and the red mitochondria had an emission peak of 585 nm. The JC-1 monomers (green fluorescence) indicate a low mitochondrial-membrane potential, whereas the JC-1 polymers (red fluorescence) indicate a high mitochondrial membrane potential. (**F**, **G**) At 35 days post-injection, vaginal smears were collected daily from the mice and stained with HE to monitor the oestrous cycle. The dynamic line charts of the mouse oestrous cycles covering 14 consecutive days in the control group (**F**) and the miR-338-3p-overexpression group (**G**) are shown

To further confirm the effect of miR-338-3p on the mouse oocyte quality and oestrous cycle in vivo, we injected AAV-EGFP (control group) and AAV-miR-338-3p (miR-338-3p-overexpression group) into the ovarian bursa of the 2-month-old female mice. Firstly, we validated the overexpression level of miR-338-3p in the ovaries of mice at 35 days post-injection by RT-qPCR, and the results showed that the overexpression level of miR-338-3p was significant (*P* < 0.0001; Fig. 7A). The number of follicles of the obtained mouse ovaries were analyzed by HE staining, and the results showed that overexpression of miR-338-3p significantly reduced the number of follicles at all levels in the mouse ovaries (Fig. 7B-D). Besides, the oocytes were harvested, stained using JC-1 dye, and observed under a laser-scanning confocal microscope. miR-338-3p overexpression was accompanied by significantly elevated oocyte staining with JC-1 (Fig. 7E), indicating that miR-338-3p overexpression decreased the oocyte mitochondrial-membrane potential and, thus, lowered the oocyte quality. Subsequently, we monitored and analysed the oestrous cycle in the injected mice. The mice in the miR-338-3p-overexpression group experienced a significantly prolonged duration of anoestrus than the mice in the control group, as well as irregular oestrous cycles (Fig. 7F, G).

## Discussion

With the emergence and improvement of ART in recent years, the rates of successful treatment and clinical pregnancies in infertile couples have been improving [19]. Nevertheless, patients with DOR that undergo IVF/ICSI-embryo transfer still face various problems including an insufficient quantity of the harvested oocytes, poor oocytes quality, and low clinical-pregnancy rate [20], which affects the treatment-success rate and causes varying degrees of physical and emotional trauma. Patients with DOR usually have relatively few sinus follicles and are insensitive to medications commonly used to induce ovulation. In addition, chronically low oestrogen levels in patients with DOR can increase their risk for developing cardio-cerebrovascular diseases and osteoporosis [1], and the disease remains idiopathic for over half of all patients with DOR. Hence, more diagnostic approaches and treatment regimens can be inspired in clinical practice by exploring the biological mechanism underlying follicular development and the occurrence of DOR. Therefore, in this study, we investigated a significantly overexpressed miR-338-3p in ovarian cortical tissue derived from POI, and explored the mechanism of miR-338-3p participating in ovarian follicle development through cell culture in vitro, neonatal mouse ovarian culture in vitro, and mouse in vivo experiments.

We performed high-throughput next-generation sequencing analysis and found that miR-338-3p expression was significantly higher in ovarian cortical tissues derived from patients with POI than in cortical tissues derived from patients with normal ovarian function. However, it remains unclear whether miR-338-3p helps regulate ovarian follicular development. Therefore, the aims of this study with human GCs and mice were to explore the relationship between the regulatory effect of miR-338-3p on granulosa cell function and ovarian hypofunction, as well as the effects of miR-338-3p on mouse follicular development, mouse oocytes, and oestrous cycles.

Previous data suggested that miRNAs are associated with POI pathogenesis. The results of a study by Nie et al. revealed that miR-23a and miR-27a expression levels were higher in serum samples from patients with POI than in serum samples from patients with normal ovarian function; miR-23a and miR-27a targeted the Smad5-activated Fas cell surface death receptor (Fas)/Fas ligand signalling pathway, thereby up-regulating the caspase 8 and caspase 3 protein levels and promoting granulosa cell apoptosis [21]. Multiple miRNAs showed significantly different expression in ovarian tissues in a chemotherapy-induced mouse model of premature ovarian failure, including miR-29a, miR-144, miR-27b, and miR-190 [22]. Specifically, miR-29a and miR-144 were significantly down-regulated and can target phospholipase A2, group IV A, cytosolic, calcium-dependent (PLA2G4A) to regulate progestogen synthesis; miR-27b and miR-190 were significantly up-regulated and can target PAPPA and CCL2 to affect the sensitivity of mouse follicles to steroid hormones. Findings by Dang et al. (2018) suggested that miR-379-5p was significantly up-regulated in GCs from patients with biochemical POI; miR-379-5p decreased the DNA-repair efficiency in GCs and suppressed granulosa cell proliferation by targeting PARP1 and XRCC6, indicating that miR-379-5p can regulate granulosa cell function and the occurrence of POI via epigenetic inheritance [23]. In addition, the results of another study demonstrated that miR-22-3p was significantly down-regulated in the serum of patients with premature ovarian failure and that it correlated negatively with serum follicle-stimulating hormone (FSH) levels; logistic-regression analysis revealed that miR-22-3p protected against premature ovarian failure; bioinformatics analysis suggested miR-22-3p target genes might be associated with apoptosis, autophagy, and tumourigenesis [24].

This study revealed that miR-338-3p expression was significantly higher in GCs derived from patients with DOR than in GCs derived from patients with normal ovarian function, which verified the high miR-338-3p-expression levels observed in ovarian cortical tissues derived from patients with POI. FISH analysis showed that miR-338-3p was expressed in the cytoplasm of primary human granulosa cells, mouse granulosa cells, and oocytes. Our results also suggested that miR-338-3p overexpression significantly suppressed the proliferation of granulosa cells, whereas miR-338-3p down-regulation significantly promoted cell proliferation. Previous studies have shown that overexpression of miR-338-3p in ovarian cancer can inhibit the growth of tumor tissue, promote apoptosis of ovarian cancer cells, and enhance the sensitivity of cancer cells to chemotherapy drugs [25]. The apoptosis of granulosa cells is correlated with the occurrence of DOR [26]. According to the above content, the result suggests that miR-338-3p may inhibit cell proliferation through high-level expression in DOR patients, leading to the occurrence of related diseases.

Some data have also suggested that miRNAs are correlated with polycystic ovary syndrome (PCOS) to a certain extent. Sørensen et al. (2016) found that the expression levels of miR-24-3p, miR-29a, miR-151-3p, and miR-574-3p in follicular fluid supernatants were significantly lower in patients with PCOS than in those from healthy controls and that miR-518f-3p expression was higher in follicular fluids from patients with classic hyperandrogenaemia than in those from patients without hyperandrogenaemia [27]. Approximately 50-70% of patients with PCOS are insulin resistant: some data showed that miR-193b, miR-194, and miR-122 were significantly up-regulated in patients with PCOS patients and impaired glucose tolerance, and functional analysis of the target genes suggested that these miRNAs might help regulate glycolipid metabolism and follicular development [28]. Another study revealed that miR-93, miR-133, and miR-223 were significantly up-regulated in adipose tissues from patients with PCOS and insulin resistance; miR-93 can down-regulate GLUT4 mRNA expression in adipose tissue and is significantly correlated with insulin resistance and glucose tolerance [29]. The results of a study by Cai et al. suggested that miR-145 was down-regulated in GCs from patients with PCOS and could suppress the mitogen‑activated protein kinase/extracellular signal‑regulated kinase signalling pathway by targeting insulin receptor substrate 1, thereby affecting granulosa cell proliferation [30]. In addition, patients with PCOS are more likely to develop ovarian hyperstimulation syndrome (OHSS) during ovulation induction due to the large number of early-stage small follicles in their ovaries. Zhao et al. found that miR-16 expression was significantly lower in the serum of patients with PCOS and severe OHSS than in the serum of control subjects with mild or no OHSS; further studies demonstrated that miR-16 might help regulate vascular endothelial growth factor synthesis and the Akt/mTOR signalling pathway; furthermore, receiver operating characteristic curve analysis suggested that the serum miR-16 level has a higher predictive value for OHSS than the Body Mass Index, serum luteinizing hormone (LH) level, and LH/FSH ratio [31].

Each ovarian follicle represents the basic functional unit of the ovary, and GCs play essential roles in follicular development: the proliferation and secretory activities of GCs directly affect the growth, development, and atresia of ovarian follicles. In this study, we first collected primary GCs from follicular fluid derived from patients with DOR and performed RT-qPCR analysis. The results suggested that miR-338-3p was expressed at significantly higher levels in GCs derived from patients with DOR than in those derived from patients with normal ovarian function. We then performed cell transfections with miR-338-3p mimics/inhibitors to manipulate miR-338-3p expression in GCs and found that miR-338-3p overexpression significantly suppressed oestradiol production by GCs and that miR-338-3p down-regulation was accompanied by significantly stronger oestradiol production capacity, suggesting that elevated miR-338-3p expression may help explain the low oestrogen levels in patients with DOR. Aromatase and oestradiol activities are crucial for the development of pre-sinus follicles into mature follicles: oestradiol can promote granulosa cell proliferation and exert positive feedback on follicular development [32]. Hence, we speculate that miR-338-3p up-regulation in GCs might lead to abnormal ovarian reserves and abnormal follicular development in patients with DOR, but further studies are required to explore these potential biological mechanisms.

Previous results showed that tissue-specific knockout of Dicer (a key enzyme for miRNA biosynthesis) in mouse varies was followed by abnormal levels of key proteins important for follicular development (including bone morphogenetic protein 15 and growth differentiation factor 9), accelerated recruitment of primordial follicles, and abnormal morphology and development of early-stage follicles [33]. TGF-β functions via autocrine or paracrine signalling and plays essential roles in regulating early follicular development [34], granulosa cell proliferation and differentiation, hormonal production, and theca cell function [35]. Previous findings suggested that some miRNAs influence in mouse follicular development by regulating the TGF-β1-Smad signalling pathway [36, 37]. Smad3-knockout GCs underwent apoptosis, leading to slower follicular development and increased follicular atresia and DOR [38]. The results of a study by Persani et al. suggested that mutations in members of the TGF-β gene family are important genetic factors for the occurrence of POI [39]. Hence, we speculate that miR-338-3p might also regulate follicular development and the ovarian reserve via the TGF-β signalling pathway. Previous data confirmed that TGF-β1 can facilitate granulosa cell proliferation and follicular maturation [40]. TGF-β1 can also improve the sensitivity of GCs to low doses of FSH [41]. Most patients with female reproductive disorders experience varying degrees of menstrual-cycle irregularities and decreases in the egg quality, and abnormalities in the menstrual cycles are one of the diagnostic criteria for POI, i.e. oligomenorrhea or amenorrhea for at least 4 months. Patients with POI that receive ART treatment experience an increased incidence of ovarian hyporesponsiveness and cycle cancellation, an insufficient quantity of harvested eggs, poor egg quality, and a reduced clinical pregnancy rate after transplantation. Abnormal menstruation caused by abnormal ovulation in patients with PCOS is manifested by irregular menstrual cycles, polymenorrhoea, oligomenorrhea, or amenorrhea, and pharmacological interventions are usually required to regulate irregular menstrual cycles. In addition, other findings have suggested that patients with PCOS and obesity usually experience impaired oocyte maturation, quality, and development potential to varying degrees during ART treatment [42].

In this study, we also explored the regulatory effect of TGFβ1 (which plays a major role in early follicular development) on miR-338-3p. The results suggested that TGF-β1 significantly down-regulated miR-338-3p expression in GCs and consequently promoted granulosa cell proliferation. We also performed in vitro culture and HE staining of neonatal mouse ovaries, and found that miR-338-3p affected the entire process of early follicular development and slowed down the primordial-to-primary follicle transition and the primary-to-secondary follicle transition. We then injected adeno associated virus vectors that drive miR-338-3p overexpression into the mouse ovarian bursae to further study the effect of miR-338-3p in vivo. We found that miR-338-3p overexpression caused a decrease in the number of follicles at all levels in mouse ovaries, and a decrease in the oocyte mitochondrial-membrane potential. The mice in the miR-338-3p-overexpression group experienced a significantly prolonged duration of anoestrus and irregular oestrous cycles. These results suggest that miR-338-3p overexpression can suppress the normal oestrous cycles in mice, leading to decreased fertility in mice.

However, our study also has some limitations. For example, the sample size of in vivo experiments by injecting adeno associated virus vectors in mouse is too small. If the sample size is conditionally expanded, the reliability of the results may be higher.

In this study, we chose primary human GCs and mice as the study subjects to examine the effect of miR-338-3p on granulosa cell function and ovarian follicular development. Our results revealed that miR-338-3p was highly expressed in ovarian cortical tissues derived from patients with POI and GCs derived from patients with DOR. Overexpression of miR-338-3p significantly suppressed the proliferation and oestrogen production of GCs and affected the early follicular development in neonatal mouse ovaries cultured in vitro. Finally, our in vivo experiments showed that miR-338-3p overexpression lowered the quality of mouse oocytes and disrupted mouse oestrous cycles.

## Summary

Previous studies have shown that microRNAs (miRNAs) play an important role in regulating ovarian function and embryonic development. In view of this, this study intends to further explore the mechanism of miRNA participating in the regulation of ovarian follicle development through in vivo and in vitro experiments. The level of miR-338-3p was significantly increased in GCs from patients with ovarian insufficiency. At the same time, the increase of miR-338-3p could inhibit the proliferation and hormone secretion of granulosa cells, slow down the development of early mouse follicles, and thus affect the occurrence of DOR. In addition, TGF-β1 can inhibit the expression of miR-338-3p in primary granulosa cells, and promote granulosa cell proliferation and follicular maturation by down regulating the expression of miR-338-3p in granulosa cells.

### Electronic supplementary material

Below is the link to the electronic supplementary material.

**Supplementary Material 1** Table S1. The primers used for the PCR.

**Supplementary Material 2** Table S2. Differential expression of miRNAs in ovarian cortical tissues from patients with normal ovarian function and primary ovarian insufficiency.

**Supplementary Material 3** Figure S1. Transfection effect of miR-338-3p mimics/inhibitor in GCs and KGN cell lines.

## Data Availability

Reasonable requests for data and materials can be obtained from the corresponding author.

## List of abbreviations

3′-UTR: 3′-untranslated region
AAV: adeno-associated virus
AKT: protein kinase B
ART: assisted reproductive technology
Bcl-2: B-cell lymphoma 2
BSA: bovine serum albumin
COC: cumulus–oocyte complex
DAPI: 4′,6-diamidino-2-phenylindole
DOR: diminished ovarian reserve
E_2_: oestradiol
EP: Eppendorf
Fas: Fas cell surface death receptor
FBS: foetal bovine serum
FISH: fluorescence in situ hybridization
FSH: follicle-stimulating hormone
GAPDH: glyceraldehyde-3-phosphate dehydrogenase
HE: haematoxylin and eosin
ICSI: intracytoplasmic sperm injection
IVF: in vitro fertilization
Ki-67: antigen identified by monoclonal antibody Ki-67
LH: luteinizing hormone
miRNA: microRNA
mRNA: messenger RNA
mTOR: mammalian target of rapamycin
NC: negative control
OD_450_: optical density at 450 nm
OHSS: ovarian hyperstimulation syndrome
p53: protein 53
PBS: phosphate-buffered saline
PBST: PBS containing 0.5% Triton X-100
PCOS: polycystic ovary syndrome
PCR: polymerase chain reaction
PI3K: phosphatidyl inositol 3-kinase
POI: premature ovarian insufficiency
pre-miRNA: precursor miRNA
pri-miRNA: primary miRNA
PVDF: polyvinylidene difluoride
RT-qPCR: reverse transcriptase-quantitative polymerase chain reaction
TBST: Tris-buffered saline with Tween-20

## References

[1] Zhang H, Liu K (2015). Cellular and molecular regulation of the activation of mammalian primordial follicles: somatic cells initiate follicle activation in adulthood. Hum Reprod Update.

[2] Devine K, Mumford SL, Wu M, DeCherney AH, Hill MJ, Propst A (2015). Diminished ovarian reserve in the United States assisted reproductive technology population: diagnostic trends among 181,536 cycles from the society for assisted Reproductive Technology Clinic Outcomes Reporting System. Fertil Steril.

[3] Xu Y, Nisenblat V, Lu C (2018). Pretreatment with coenzyme Q10 improves ovarian response and embryo quality in low-prognosis young women with decreased ovarian reserve: a randomized controlled trial. Reprod Biol Endocrinol.

[4] Jiao X, Ke H, Qin Y, Chen ZJ (2018). Molecular Genetics of premature ovarian insufficiency. Trends Endocrinol Metab.

[5] Gurtcheff SE, Klein NA (2011). Diminished ovarian reserve and infertility[J]. Clin Obstet Gynecol.

[6] Hipp HS, Kawwass JF (2019). Discordant ovarian reserve testing: what matters most. Fertil Steril.

[7] Bunnewell SJ, Honess ER, Karia AM, Keay SD, Al Wattar BH, Quenby S (2020). Diminished ovarian reserve in recurrent pregnancy loss: a systematic review and meta-analysis. Fertil Steril.

[8] Ambros V (2004). The functions of animal microRNAs[J]. Nature.

[9] Bartel DP (2004). MicroRNAs: genomics, biogenesis, mechanism, and function[J]. Cell.

[10] Saliminejad K, Khorram Khorshid HR, Soleymani Fard S (2019). An overview of microRNAs: Biology, functions, therapeutics, and analysis methods[J]. J Cell Physiol.

[11] Correia de Sousa M, Gjorgjieva M, Dolicka D et al. Deciphering miRNAs’ Action through miRNA Editing[J]. Int J Mol Sci, 2019,20(24). 10.3390/ijms20246249 PMC6941098 31835747

[12] Buchan JR, Parker R (2007). Molecular biology. The two faces of miRNA[J]. Science.

[13] Liu W, Niu Z, Li Q, Pang RT, Chiu PC, Yeung WS (2016). MicroRNA and embryo implantation. Am J Reprod Immunol.

[14] Dai A, Sun H, Fang T (2013). MicroRNA-133b stimulates ovarian estradiol synthesis by targeting Foxl2[J]. FEBS Lett.

[15] Yang S, Wang S, Luo A (2013). Expression patterns and regulatory functions of microRNAs during the initiation of primordial follicle development in the neonatal mouse ovary[J]. Biol Reprod.

[16] Takagi K, Yamada T, Miki Y, Umegaki T, Nishimura M, Sasaki J (2007). Histological observation of the development of follicles and follicular atresia in immature rat ovaries. Acta Med Okayama.

[17] Byers SL, Wiles MV, Dunn SL, Taft RA (2012). Mouse estrous cycle identification tool and images. PLoS ONE.

[18] Scholzen T, Gerdes J (2000). The Ki-67 protein: from the known and the unknown. J Cell Physiol.

[19] Donadeu FX, Schauer SN, Sontakke SD (2012). Involvement of miRNAs in ovarian follicular and luteal development. J Endocrinol.

[20] Ma T, Jiang H, Gao Y (2011). Microarray analysis of differentially expressed microRNAs in non-regressed and regressed bovine corpus luteum tissue; microRNA-378 may suppress luteal cell apoptosis by targeting the interferon gamma receptor 1 gene. J Appl Genet.

[21] Nie M, Yu S, Peng S (2015). miR-23a and miR-27a promote human granulosa cell apoptosis by targeting SMAD5. Biol Reprod.

[22] Kuang H, Han D, Xie J (2014). Profiling of differentially expressed microRNAs in premature ovarian failure in an animal model. Gynecol Endocrinol.

[23] Dang Y, Wang X, Hao Y (2018). MicroRNA-379-5p is associate with biochemical premature ovarian insufficiency through PARP1 and XRCC6. Cell Death Dis.

[24] Dang Y, Zhao S, Qin Y (2015). MicroRNA-22-3p is down-regulated in the plasma of Han Chinese patients with premature ovarian failure. Fertil Steril.

[25] Niu Q, Liu Z, Gao J (2019). MiR-338-3p enhances ovarian Cancer cell sensitivity to cisplatin by downregulating WNT2B[J]. Yonsei Med J.

[26] Hussein MR (2005). Apoptosis in the ovary: molecular mechanisms[J]. Hum Reprod Update.

[27] Sørensen AE, Wissing ML, Englund AL (2016). MicroRNA Species in Follicular Fluid associating with polycystic ovary syndrome and related intermediary phenotypes. J Clin Endocrinol Metab.

[28] Jiang L, Huang J, Chen Y, Yang Y, Li R, Li Y, Chen X, Yang D (2016). Identification of several circulating microRNAs from a genome-wide circulating microRNA expression profile as potential biomarkers for impaired glucose metabolism in polycystic ovarian syndrome. Endocrine.

[29] Chen YH, Heneidi S, Lee JM (2013). miRNA-93 inhibits GLUT4 and is overexpressed in adipose tissue of polycystic ovary syndrome patients and women with insulin resistance. Diabetes.

[30] Cai G, Ma X, Chen B (2017). MicroRNA-145 negatively regulates cell proliferation through targeting IRS1 in isolated ovarian granulosa cells from patients with polycystic ovary syndrome. Reprod Sci.

[31] Zhao C, Liu X, Shi Z (2015). Role of serum miRNAs in the prediction of ovarian hyperstimulation syndrome in polycystic ovarian syndrome patients. Cell Physiol Biochem.

[32] Britt KL, Saunders PK, McPherson SJ (2004). Estrogen actions on follicle formation and early follicle development. Biol Reprod.

[33] Lei L, Jin S, Gonzalez G (2010). The regulatory role of Dicer in folliculogenesis in mice[J]. Mol Cell Endocrinol.

[34] Moore RK, Shimasaki S (2005). Molecular biology and physiological role of the oocyte factor, BMP-15[J]. Mol Cell Endocrinol.

[35] Spicer LJ, Aad PY, Allen DT (2008). Growth differentiation factor 9 (GDF9) stimulates proliferation and inhibits steroidogenesis by bovine theca cells: influence of follicle size on responses to GDF9[J]. Biol Reprod.

[36] Yao G, Yin M, Lian J (2010). MicroRNA-224 is involved in transforming growth factor-beta-mediated mouse granulosa cell proliferation and granulosa cell function by targeting Smad4[J]. Mol Endocrinol.

[37] Smith AL, Iwanaga R, Drasin DJ (2012). The miR-106b-25 cluster targets Smad7, activates TGF-β signaling, and induces EMT and tumor initiating cell characteristics downstream of Six1 in human breast cancer[J]. Oncogene.

[38] Tomic D, Miller KP, Kenny HA (2004). Ovarian follicle development requires Smad3[J]. Mol Endocrinol.

[39] Persani L, Rossetti R, Cacciatore C (2011). Genetic defects of ovarian TGF-B-like factors and premature ovarian failure[J]. J Endocrinol Invest.

[40] Fang L, Chang HM, Cheng JC (2014). TGF-β1 downregulates StAR expression and decreases progesterone production through Smad3 and ERK1/2 signaling pathways in human granulosa cells[J]. J Clin Endocrinol Metab.

[41] Zachow RJ, Weitsman SR, Magoffin DA (1999). Leptin impairs the synergistic stimulation by transforming growth factor-beta of follicle-stimulating hormone-dependent aromatase activity and messenger ribonucleic acid expression in rat ovarian granulosa cells[J]. Biol Reprod.

[42] Liu T, Liu D, Song X, Qu J, Zheng X, Li J, Yang R, Yang S, Zhang X, Wang H, Yan L, Ma C, Li R, Yan J, Qiao J (2021). Lipid metabolism was Associated with oocyte in vitro maturation in women with polycystic ovarian syndrome undergoing unstimulated natural cycle. Front Cell Dev Biol.

