# Supplementary figures and images for: MicroRNA-338-3p helps regulate ovarian function by affecting granulosa cell function and early follicular development
Source: J Ovarian Res. 2023 Aug 26;16:175. doi: 10.1186/s13048-023-01258-3 (PMC10463366; doi:10.1186/s13048-023-01258-3)

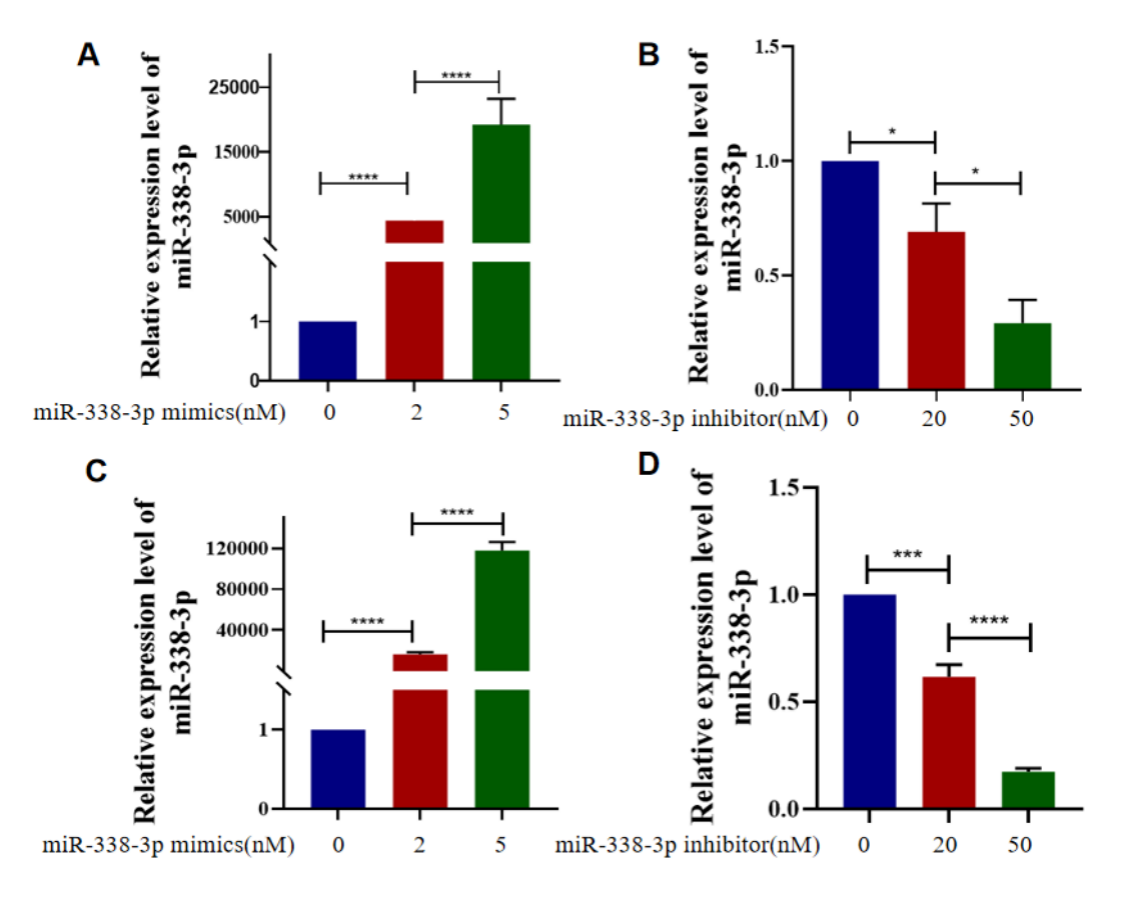

Supplement: Supplementary file 3 — Supplementary Material 3 Figure S1. Transfection effect of miR-338-3p mimics/inhibitor in GCs and KGN cell lines. [file 13048_2023_1258_MOESM3_ESM.png]
